# Understanding the Respite for All Model: Experiences of People Living With Dementia, Caregivers, and Volunteers

**DOI:** 10.1093/geroni/igaf122.3866

**Published:** 2025-12-31

**Authors:** Eleanor Hummel, Camille Murray, Siyue Fang, Karl Umble, Erin Kent

**Affiliations:** University of North Carolina at Chapel Hill, Chapel Hill, North Carolina, United States; University of North Carolina at Chapel Hill, Chapel Hill, North Carolina, United States; University of North Carolina at Chapel Hill, Chapel Hill, North Carolina, United States; University of North Carolina at Chapel Hill, Chapel Hill, North Carolina, United States; University of North Carolina at Chapel Hill, Chapel Hill, North Carolina, United States

## Abstract

Respite care is a long-term service and support (LTSS) that provides instrumental support to family caregivers. The volunteer-run Respite for All (RFA) model has sought to increase access to LTSS. There are over fifty iterations of the model across fifteen states. Our study aimed to qualitatively evaluate the RFA model. During the first phase, the RFA model was characterized through semi-structured Key Informant Interviews (KIIs) with RFA leaders. After characterizing the RFA model, semi-structured in-depth interviews (IDIs) were conducted to understand the experiences of caregivers, PLWD, and volunteers with one iteration of the model. Thematic analysis was performed. KIIs were conducted with RFA leaders (n = 7), and IDIs were conducted with PLWD (n = 3), caregivers (n = 5), and volunteers (n = 5). A logic model was used to organize program inputs, activities, outputs, and impacts. Program leaders reported distinctive characteristics about the RFA model, including high replicability, engagement, and the ability to deliver person-centered care. Program experiences of PLWD, caregivers, and volunteers were reported to be overwhelmingly positive and included increased feelings of well-being, acceptance, purpose, social support, engagement, and community. Facilitators to accessing programs included: word-of-mouth, diverse volunteers, geographic proximity, feedback, inclusivity, other program supports, strong connections with caregivers, and honoring and remembering participants. Barriers to access were also described: stigma and cultural beliefs around respite care and dementia, program capacity and awareness, distrust, geographic limitations, lack of diversity, dementia progression, and cost. This study provides a foundation for future research on the RFA model, an innovative approach to respite care.

